# Supplementary material for: Non-Isocyanate Synthesis of Aliphatic Polyurethane by BiCl3-Catalyzed Transurethanization Polycondensation
Source: Polymers (Basel). 2024 Apr 18;16(8):1136. doi: 10.3390/polym16081136 (PMC11053453; doi:10.3390/polym16081136)
Supplement: Supplementary file 1 [file polymers-16-01136-s001.zip › polymers-2964436-supplementary.pdf]

polymers

MDPI

Supplementary Materials for

Non-isocyanate synthesis of aliphatic polyurethane by $BiCl_3$-catalyzed transurethanization polycondensation

Bungo Ochiai and Yuriko Kobayashi

Graduate School of Science and Engineering, Yamagata University; ochiai@yz.yamagata-u.ac.jp

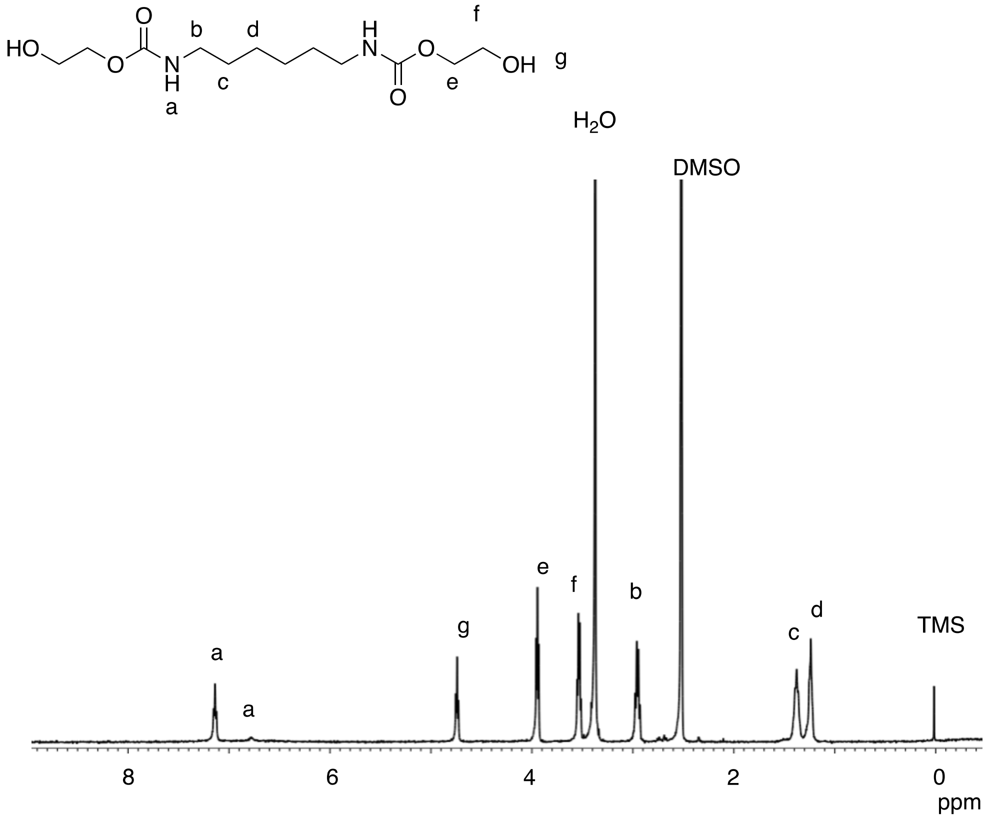

Figure S1. ^{1}H NMR spectrum of BHU6 (d_6-DMSO, 400 MHz).

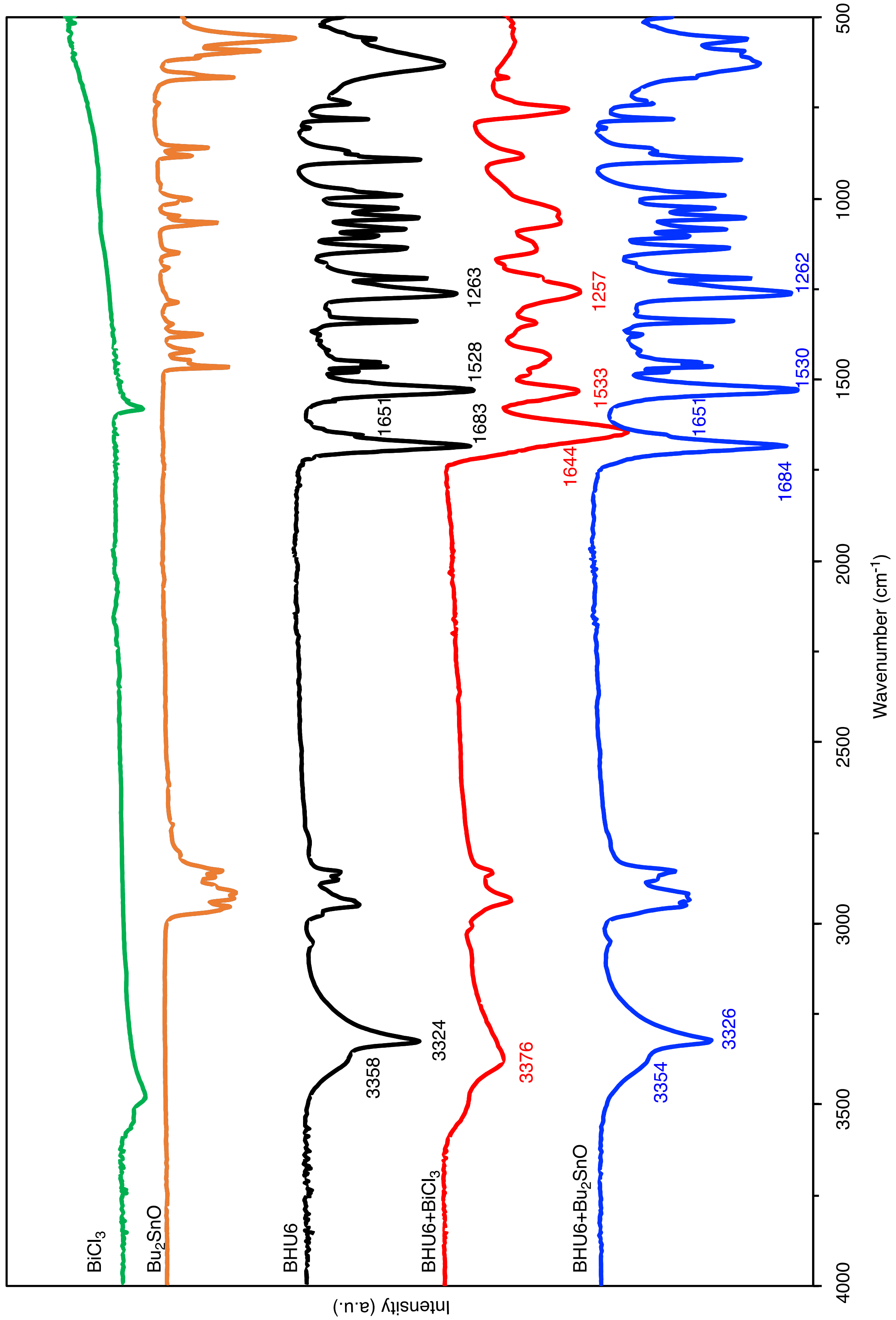

Figure S2. Full-range IR spectra for Figure 1.

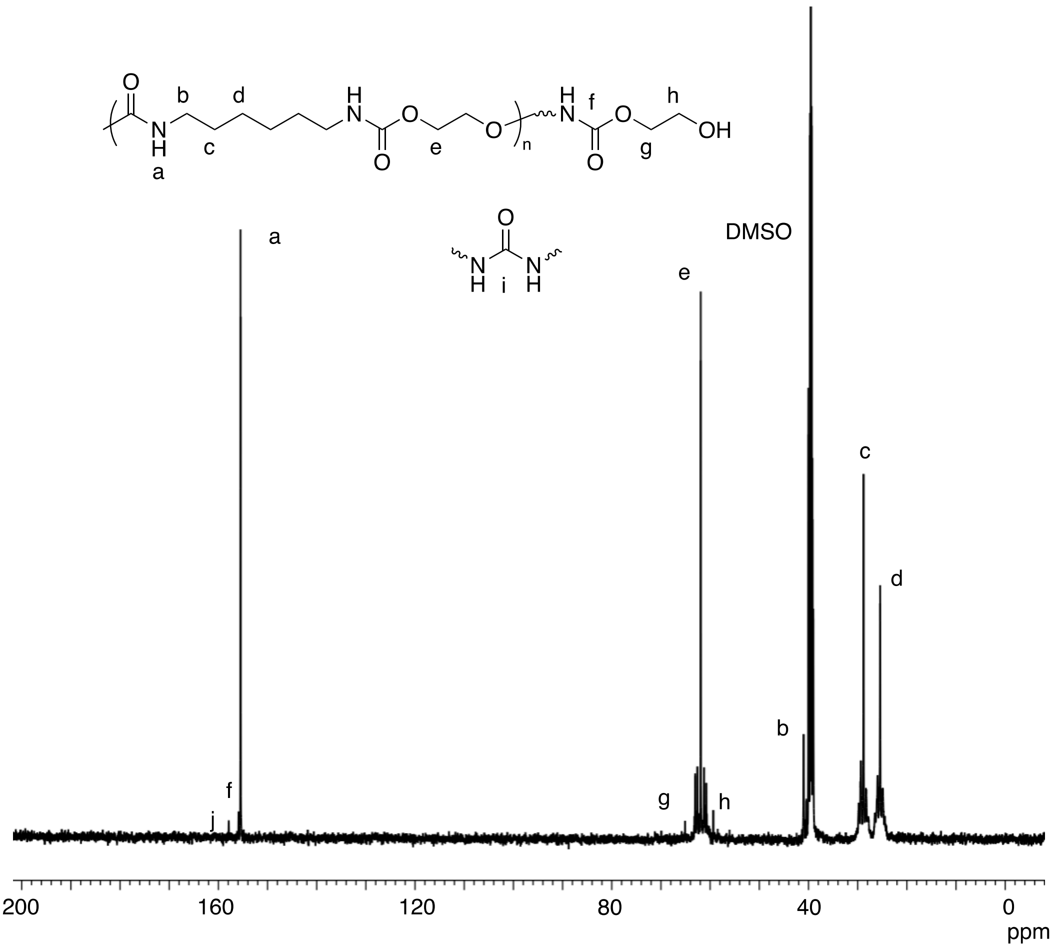

Figure S3. ^{13}C NMR spectrum of [6,2]-polyurethane obtained by self-condensation of BHU6 at 150 °C for 5 h in xylene using $BiCl_3$ (d_6-DMSO, 125 MHz)).

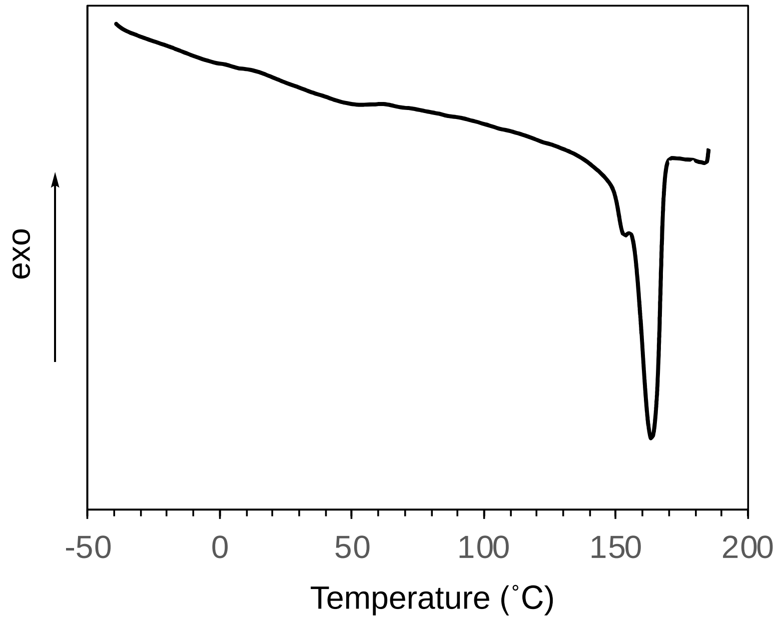

Figure S4. DSC curve of [6,2]-polyurethane (10˚C/min, N_2, second heating scan).

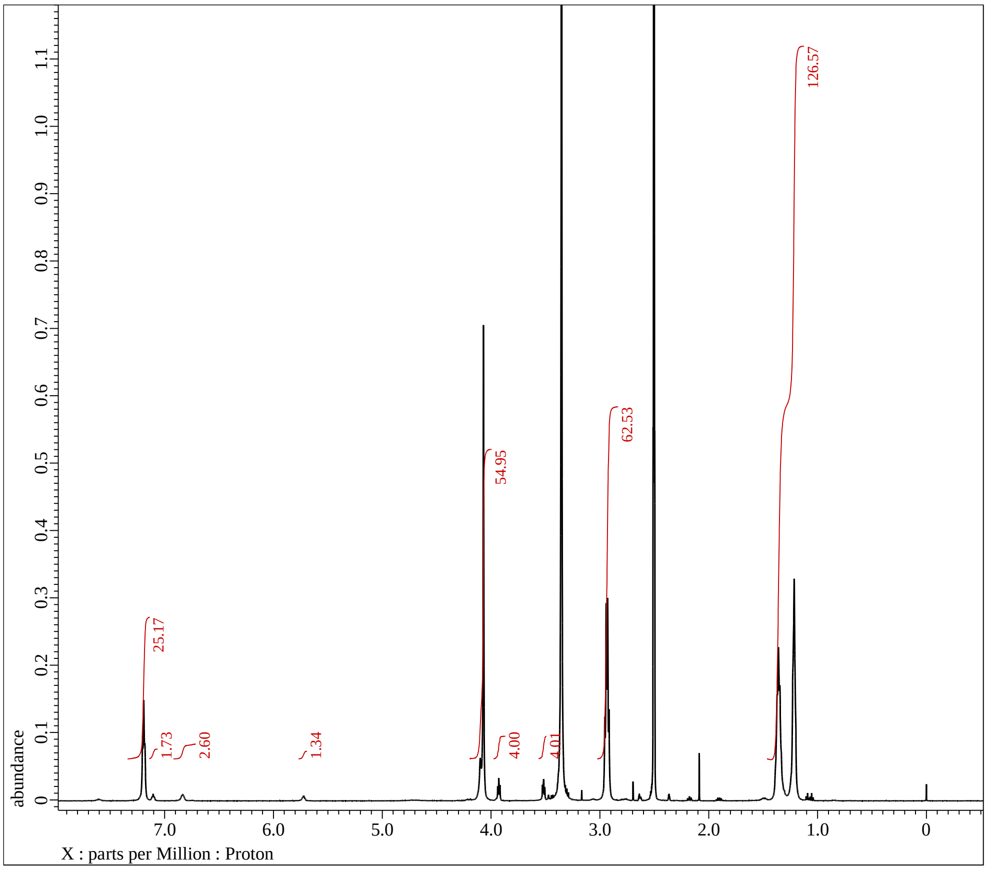

Figure S5. ^{1}H NMR spectrum of [6,2]-polyurethane obtained in Entry 1 in Table 4..

Calculation of M_{nNMR}

Molecular weight of unit = 230.26

Molecular weight of terminal group = 292.33

Degree of polymerization (–1): (62.53-4)/4 = 14.6

M_{nNMR} = 230.26*14.6+292.36 = 3661.6

Calculation of Urethane/Urea ratio

Urea proton: 1.34

Urethane protons: 25.17+1.73+2.60 = 29.5

Urethane/Urea = 96/4

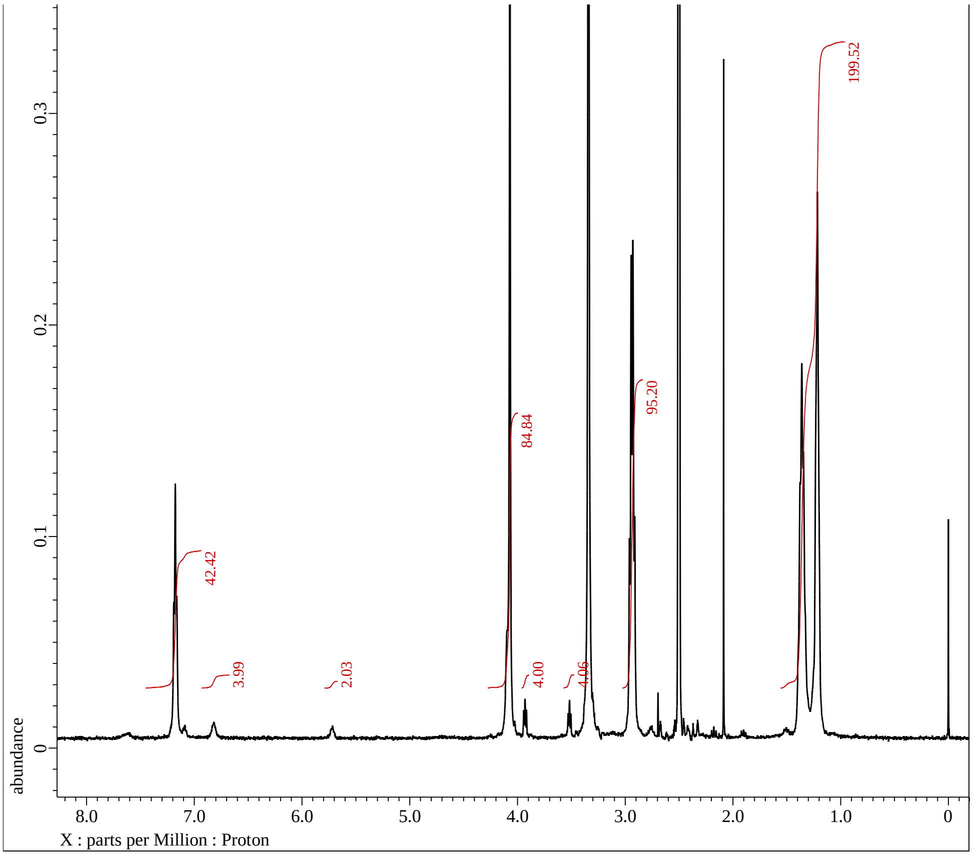

Figure S6. ^{1}H NMR spectrum of [6,2]-polyurethane obtained in Entry 2 in Table 4.

Calculation of M_{nNMR}

Degree of polymerization (–1): (95.20-4)/4 = 22.8

M_{nNMR} = 230.26*22.8+292.36 = 5542.3

Calculation of Urethane/Urea ratio

Urea proton: 2.03

Urethane protons: 42.42+3.99 = 46.41

Urethane/Urea = 96/4

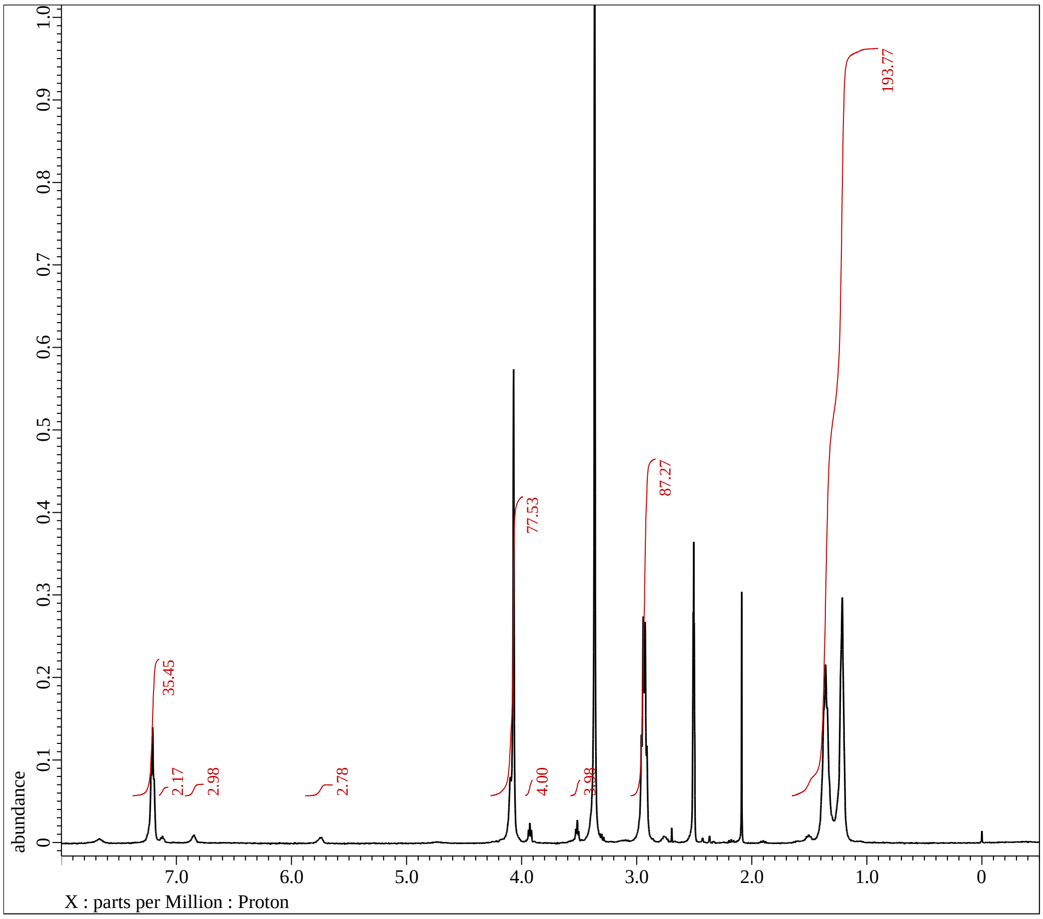

Figure S7. ^{1}H NMR spectrum of [6,2]-polyurethane obtained in Entry 3 in Table 4.

Calculation of M_{nNMR}

Degree of polymerization (−1): (87.27-4)/4 = 20.8

M_{nNMR} = 230.26*20.8+292.36 = 5085.8

Calculation of Urethane/Urea ratio

Urea proton: 2.78

Urethane protons: 35.43+2.17+2.98 = 40.6

Urethane/Urea = 94/6
